# Correlation Between Retinal Microstructure Detected by Optical Coherence Tomography and Best Corrected Visual Acuity in Diabetic Retinopathy Macular Edema

**DOI:** 10.3389/fendo.2022.831909

**Published:** 2022-06-01

**Authors:** Siying Li, Rui Hua, Zuoqian Jing, Lele Huang, Lei Chen

**Affiliations:** ^1^Department of Ophthalmology, The First Affiliated Hospital of Jiamusi University, Jiamusi, China; ^2^Department of Ophthalmology, The First Affiliated Hospital of China Medical University, Shenyang, China

**Keywords:** optical coherence tomography, photoreceptor layer, external limiting, central foveal thickness, diabetic retinopathy macular edema

## Abstract

**Objective:**

This study aimed to investigate the correlation between best corrected visual acuity (BCVA) and retinal microstructural parameters detected by optical coherence tomography (OCT) in diabetic retinopathy macular edema (DRME).

**Methods:**

Thirty-nine patients (64 eyes) with DRME were enrolled in this study. These patients underwent OCT to measure the fracture distance of the external limiting membrane (ELM), junction between the inner and outer segments (IS/OS), central foveal thickness (CFT), and edema layer. The correlation between the above parameters and BCVA was discussed.

**Results:**

CFT and the fracture distances of the ELM and IS/OS layers were negatively correlated with BCVA (p<0.05 for all). There was significant difference in Logarithm of the minimum angle of resolution (LogMAR) BCVA among patients with inner retinal edema, outer retinal edema, and mixed retinal edema (F = 5.57, p = 0.01). The LogMAR BCVA of inner retinal edema was the lowest (p < 0.05), and the LogMAR BCVA of outer retinal edema and mixed retinal edema were comparable (p > 0.05).

**Conclusion:**

In eyes with DRME, thin CFT, intact ELM and IS/OS layers, and edema in inner retina is closely correlated with good BCVA.

## 1 Introduction

As a serious complication of diabetes, diabetic retinopathy macular edema (DRME) is a common eye disease that causes blindness and the main cause of vision loss in diabetic retinopathy (DR) (1). DR can destroy the blood-retina barrier, and the intravascular components such as proteins, lipids and inflammatory factors leak out the barrier, changing osmotic pressure and protein gradient (2). Metabolism of cell is thus affected, resulting in cellular edema or shrinking, ion disorder, membrane depolarization. These pathologic conditions lead to changes of microenvironment of the retina, resulting in disruption of the retina especially the outer retina (3, 4). Clinical examination of DRME mainly depends on ophthalmoscopy, fundus fluorescein angiography, a slit lamp, and optical coherence tomography (OCT). Compared with other inspection methods, OCT can be used not only for tomography and interlayer positioning, but also for quantitative measurement; therefore, it is widely used in the clinical examination of retinal diseases such as branch retinal vein occlusion, chorioretinitis, and retinal detachment. This study was designed to inspect the retinal microstructure in DRME with OCT and analyze the correlation between retinal microstructure and best corrected visual acuity (BCVA) to demonstrate the pathological characteristics and provide a reference for treatment of DRME.

## 2 Subjects and Methods

### 2.1 Subjects

Between September 2017 and March 2019, 39 patients (64 eyes) were diagnosed with DRME in our hospital and enrolled in this study. The average age of these patients was 58.6 ± 13.5 years. These patients included 19 males (48.72%) and 20 females (51.28%). Two patients had type I diabetes mellitus (5.13%), and 37 patients had type II diabetes mellitus (94.87%). The diseased eyes included 33 right eyes (51.56%) and 31 left eyes (48.44%). The course of disease was 11.4 ± 6.3 years. Inclusion criteria: (1) DRME was diagnosed according to the Early Treatment of Diabetic Retinopathy Study (ETDRS) evaluation criteria (5); and (2) in OCT images, the central foveal thickness (CFT) was ≥250 μm, and the junction between inner and outer segments (IS/OS) was involved. Exclusion criteria: (1) history of intraocular surgery; (2) refractive stromal opacity such as keratoleukoma and grade V sclerotic nuclei cataract; (3) macular edema associated with epimacular membrane, vitreoretinal proliferation, uveitis, and posterior vitreous detachment; (4) history of undergoing retinal laser photocoagulation, peribulbar vitreous cavity surgery, or injection of hormone drugs within 2 years.

### 2.2 Methods

#### 2.2.1 Treatment

Two doctors with more than 3 years’ experience in ophthalmic examination performed OCT and recorded the BCVA in 39 patients. The Heidelberg SPECTRALIS optical coherence tomoscanner (Spectarlis HRA + OCT; Heidelberg Engeneering, Heidelberg, Germany) with image resolution of more than 16 dB was used. The diseased macular area was scanned in four directions (0°, 45°, 90°, and 135°), with the fovea as the center and 1 mm as the radius. The distance of the accumulated fracture zone of the ELM layer and IS/OS layer was measured on the scanogram, and the mean was calculated. The CFT of each eye was measured and classified as follows: inner retinal edema, the inner five layers of the retina were affected; outer retinal edema, the outer five layers of the retina were affected; and mixed retinal edema, the inner and outer layers of the retina were affected. BCVA was obtained by optometry.

### 2.3 Statistical Analysis

The data analysis was conducted using SPSS 19.0. Measurement data, such as LogMAR BCVA and CFT, were evaluated using one-way analysis of variance (ANOVA). The correlation between data, such as the correlation between CFT and LogMAR BCVA, was evaluated using Pearson correlation. P < 0.05 was considered statistically significant.

## 3 Results

### 3.1 Correlation Between CFT and BCVA

**Figure 1:**
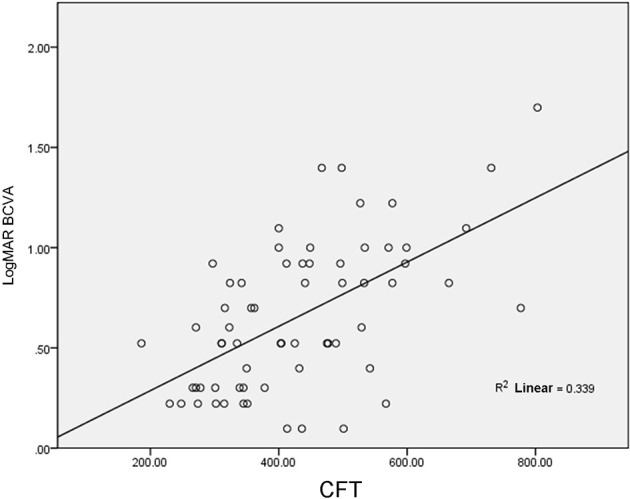
Correlation between CFT and LogMAR BCVA.

In 39 patients (64 eyes), LogMAR BCVA was 0.66 ± 0.37 and CFT was 430.48 ± 135.01 μm; therefore, CFT was positively correlated with LogMAR BCVA (r = 0.58, p <0.001, **Figure 1**).

### 3.2 Correlation Between Fracture Distance of ELM Layer and BCVA

**Figure 2:**
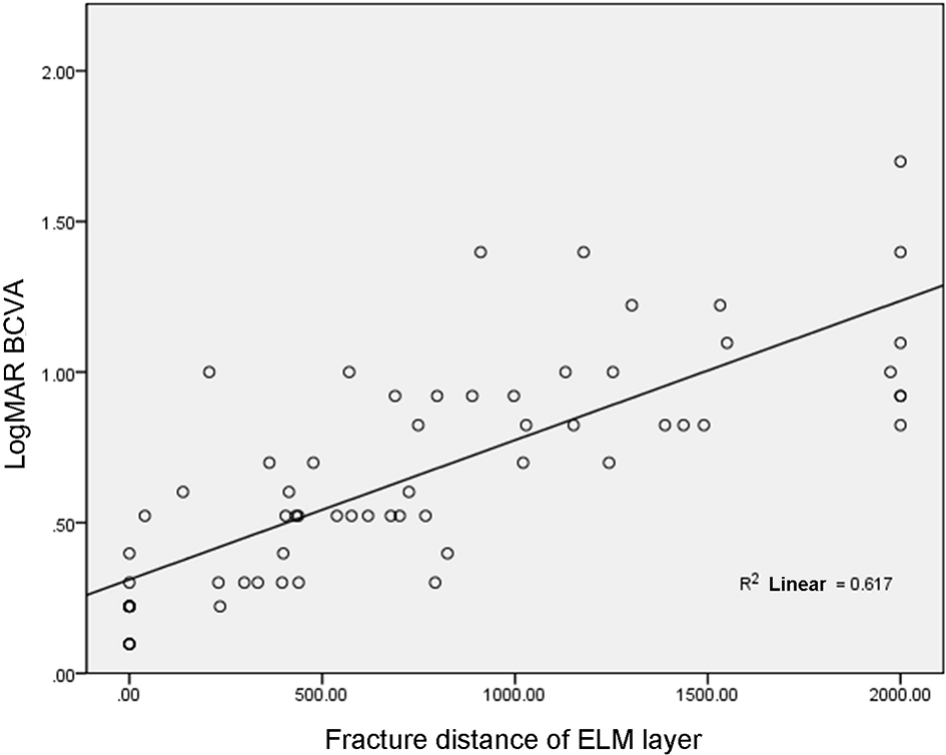
Correlation between fracture distance of ELM layer and LogMAR BCVA.

In 39 patients (64 eyes), the fracture distance of the ELM layer was 746.19 ± 631.01 μm, which was positively correlated with LogMAR BCVA (r = 0.79, p< 0.001, **Figure 2**).

### 3.3 Correlation Between Fracture Distance of IS/OS Layer and BCVA

**Figure 3:**
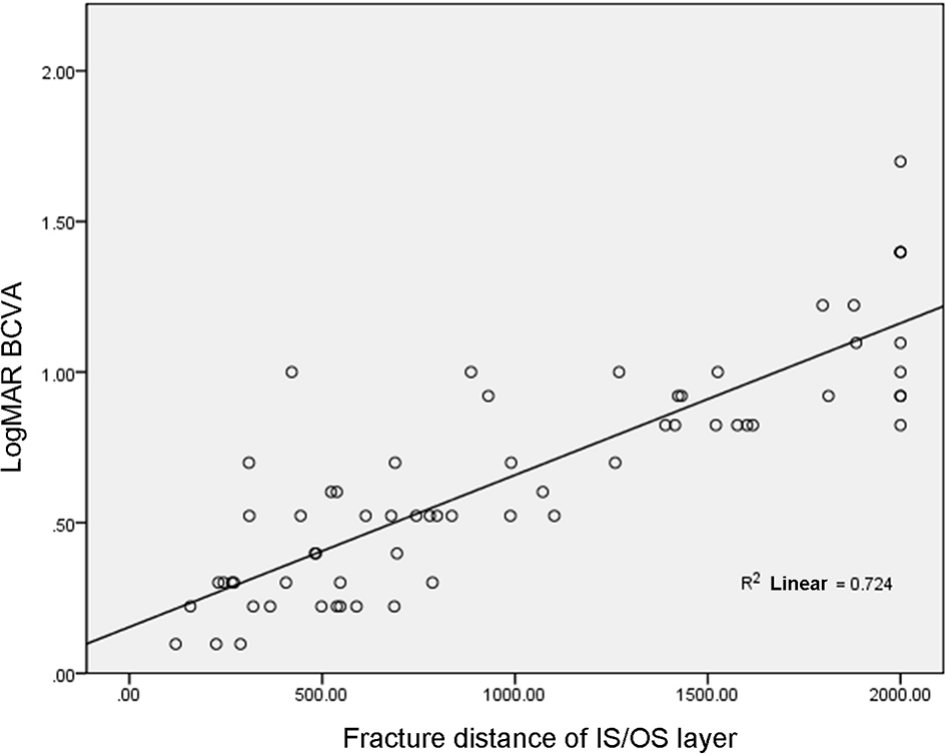
Correlation between fracture distance of IS/OS layer and LogMAR BCVA.

In 39 patients (64 eyes), the fracture distance of the IS/OS layer was 997.00 ± 627.01 μm, which was positively correlated with LogMAR BCVA (r = 0.85, p< 0.001, **Figure 3**).

### 3.4 Correlation Between Edema Layer and BCVA

**Figure 4:**
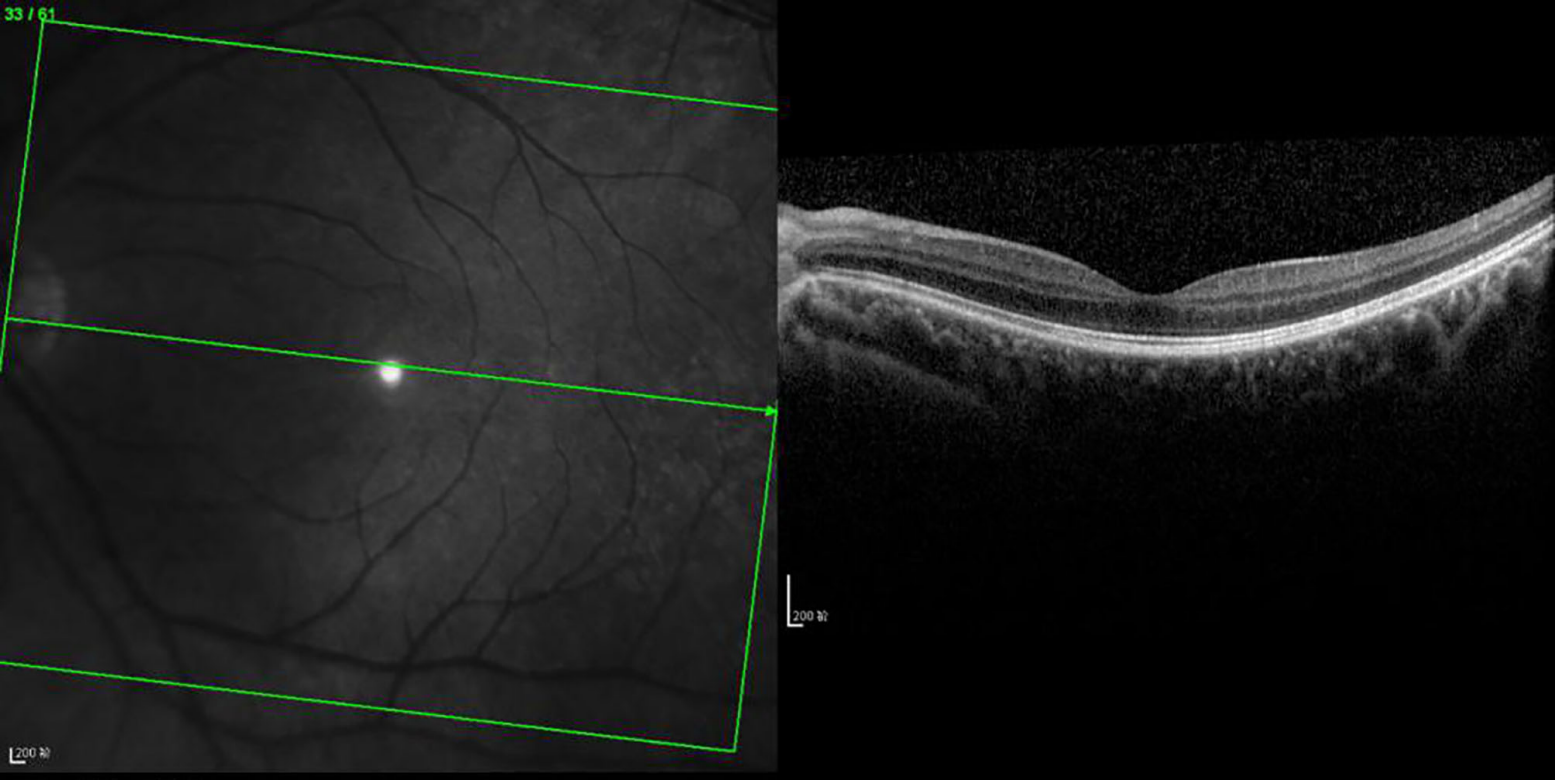
OCT scanning of the ocular fundus in an individual without DRME. There is no edema in the macular area, the CFT value is 231 μm, and the ELM layer and IS/OS layer are continuous.

**Figure 5:**
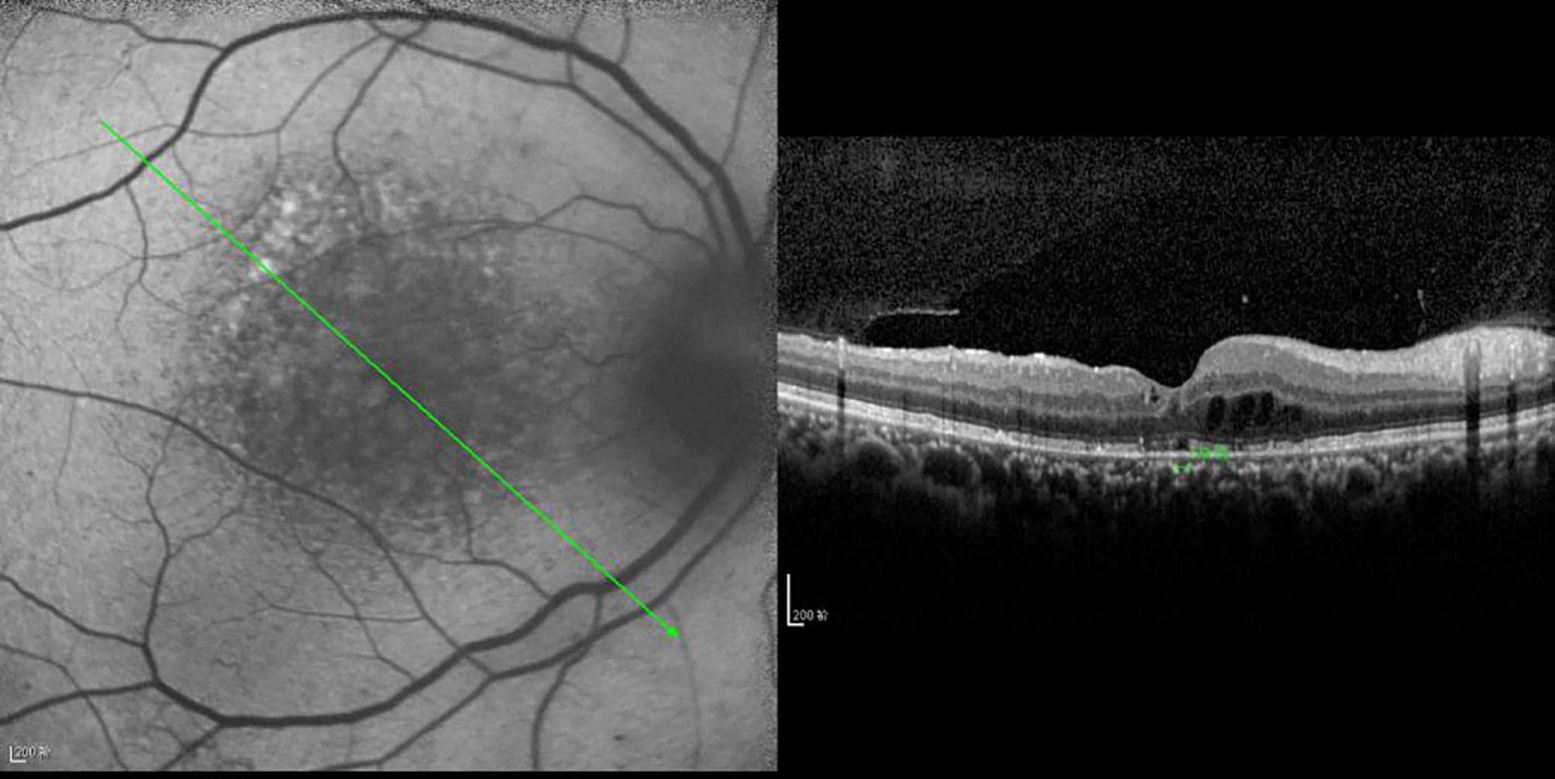
OCT scanning of the ocular fundus in patients with DRME. The CFT value is 397 μm, the ELM layer is continuous, the fracture distance of the IS/OS layer is 139 μm, and there is outer retinal edema.

**Figure 6:**
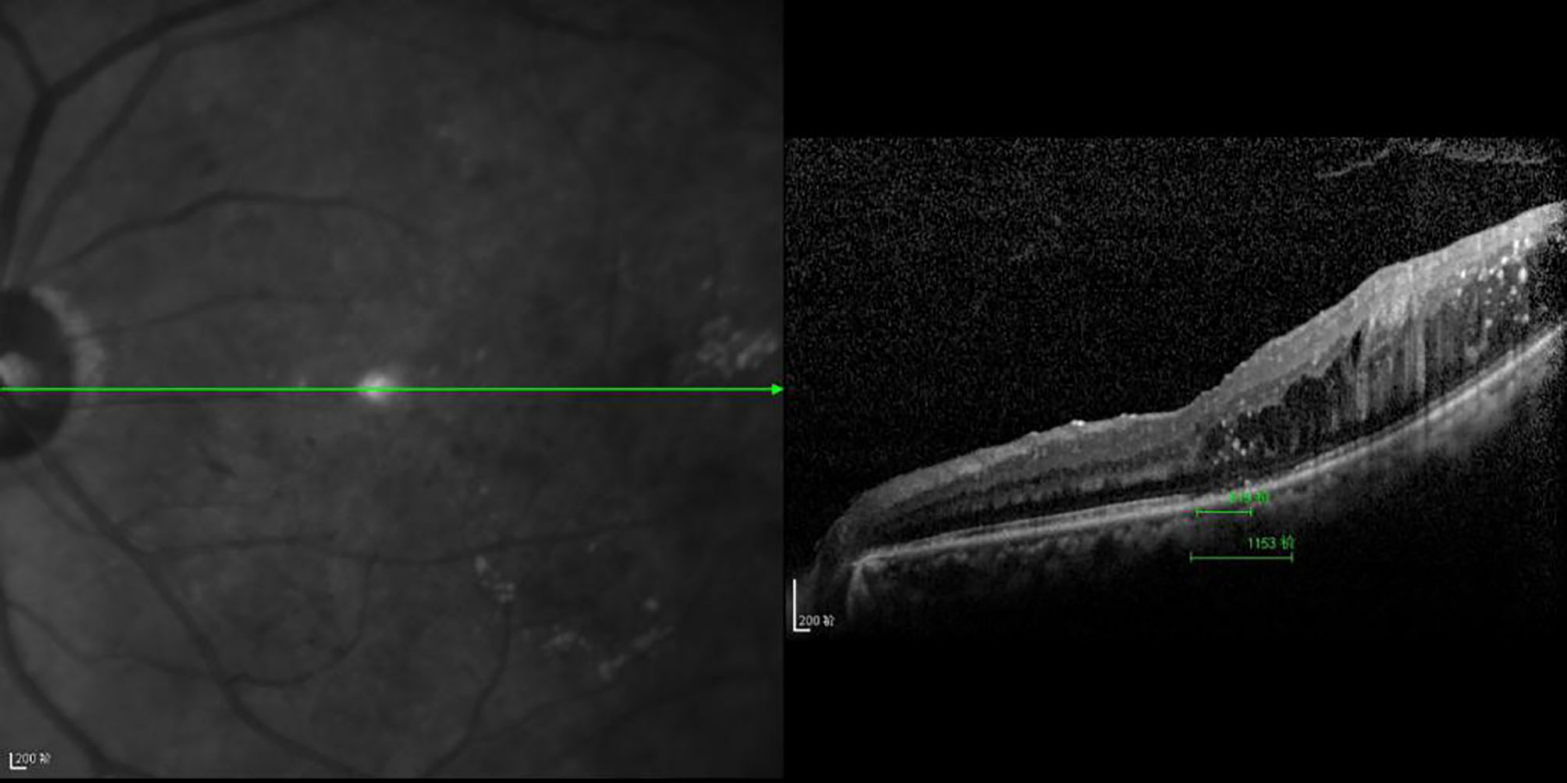
The CFT value is 519 μm, the ELM layer is continuous, the fracture distance of the IS/OS layer is 1,153 μm, and there is outer retinal edema.

**Figure 7:**
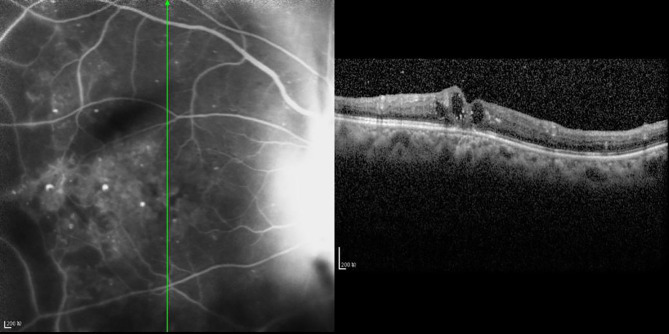
The CFT value is 437 μm, the fracture distance of the ELM layer is 897 μm, the fracture distance of the IS/OS layer is 977 μm, and there is mixed retinal edema.

**Figure 8:**
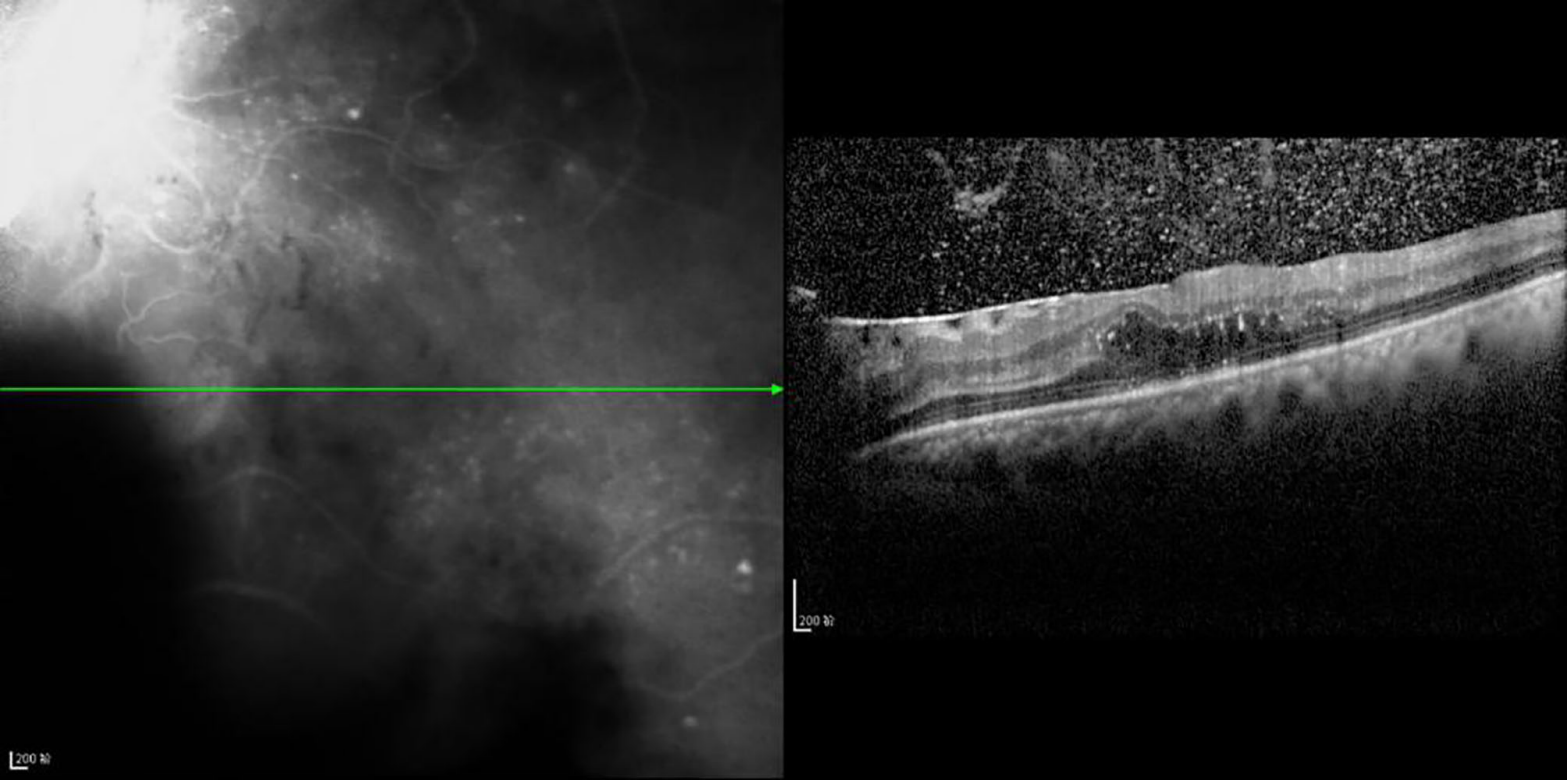
The CFT value is 536 μm, the fracture distance of the ELM layer is 1,189 μm, the fracture distance of the IS/OS layer is >2,000 μm, and there is outer retinal edema.

**Figure 9:**
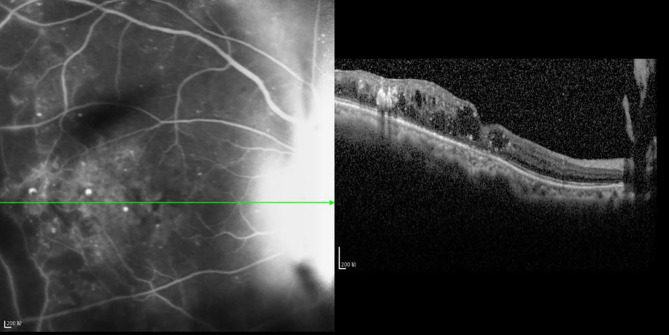
The CFT value is 352 μm, the fracture distance of the ELM and IS/OS layers is >2,000 μm, and there is mixed retinal edema. R^2^ Linear.

There was inner, outer, and mixed retinal edema in 18, 29, and 17 eyes, respectively, and the difference in LogMAR BCVA was statistically significant ([0.43 ± 0.24] vs. [0.72 ± 0.35] vs. [0.79 ± 0.43], F = 5.57, p = 0.01). The LogMAR BCVA of inner retinal edema was smaller than that of outer retinal edema (p < 0.05), the LogMAR BCVA of inner retinal edema was smaller than that of mixed retinal edema (p < 0.05), and there was no significant difference in LogMAR BCVA between outer retinal edema and mixed retinal edema (p > 0.05, **Figures 4**–**9**).

## 4 Discussion

In related studies, in OCT examination, the correlation between CFT and LogMAR BCVA was different in DRME. For example, studies have revealed that there was a positive correlation between the two, and r values ranged from 0.39 to 0.79 (6–9). A study also revealed that CFT was negatively correlated with LogMAR BCVA in DRME (r = −0.23) (10). This study revealed that there was a negative correlation between CFT and BCVA, and the higher the CFT, the lower the BCVA is. CFT and LogMAR BCVA were used as baseline values and compared. It was found that BCVA increased with an increase in CFT in 17% of the eyes and decreased with a decrease in CFT in 16% of the eyes. However, this contradicts the overall results, and the reason may be that there were different degrees of ELM layer and/or IS/OS layer fracture in the eyes of the patients included in this study, which affected BCVA. Shen et al. (11) reached a similar conclusion, namely the severity of macular edema did not match BCVA; however, the integrity of photoreceptors plays a very important role in the limited vision in DRME.

In addition, this study revealed that the fracture distance of both the ELM layer and the IS/OS layer was statistically and negatively correlated with BCVA; this result is consistent with those reported in the related literature (12–14). However, most of the literature reflects the relationship between the integrity of the ELM layer and/or IS/OS layer with BCVA by classifying variables. For example, the continuity of the ELM layer and/or IS/OS layer was artificially divided into two groups (positive and negative) or three fractured groups, and then the difference in BCVA among different groups was compared. In this study, quantitative processing of the ELM layer and IS/OS layer was carried out to calculate the fracture distance in four directions, making the results of the ELM layer and IS/OS layer more reliable. The results of the present study revealed that the fracture distance of the ELM layer (746.19 μm) was shorter than that of the IS/OS layer (997.00 μm), which can provide a reference for the quantitative relationship between ELM layer fracture and the IS/OS layer. Regarding the correlation between the ELM layer and the IS/OS layer and visual acuity, Maheshwary et al. highlight that whether the IS/OS layer is damaged is a risk factor for BCVA (12). The present study supports the conclusion of the literature in that compared with the ELM layer, the IS/OS layer can affect visual acuity, namely compared with BCVA, the damage or fracture degree of the IS/OS layer has a stronger correlation with BCVA (r = 0.851 > r = 0.786).

The ELM of photoreceptor cells connecting Muller cells has a barrier function (15). Therefore, when Muller cells and glial cells become diseased, such as through degeneration or edema, the barrier function of the ELM layer is destroyed, causing large molecules, such as inflammatory cells in the blood, to be transferred to the outer retina through the damaged ELM layer, changing the protein concentration. Consequently, the osmotic pressure balance of the outer retina is damaged, the photoreceptor cells are destroyed, and the function of nerve signal transduction is reduced (14). The inner segment of the retinal photoreceptor is rich in mitochondria, and its damage and loss are considered to be a sign of decreased photoreceptor function, which is also associated with a decrease in BCVA (16). The ELM is a complex consisting of extended myoid photoreceptor cells and the foot plate of Muller cells, which is involved in the maturation and polarization of photoreceptor cells (17). A study highlights that the incompleteness of the IS/OS layer structure is closely related to the imperfection of the ELM layer (11), and ELM integrity recovery is earlier than IS/OS layer (18). Therefore, in patients with DRME, after treatment, the recovery of ELM is regarded as the key to BCVA recovery.

In OCT, when it scans the abnormal cells in the retinal pigment epithelial (RPE) layer, it indicates that macrophages in the RPE layer are activated or subjected to metaplasia or phagocytosis. The damaged RPE layer can indirectly reduce the activity or function of photoreceptor cells (19), which proves that the IS/OS layer is closely related to BCVA. A previous study revealed that compared with the other types, the damage to the integrity of ELM and IS/OS was mostly presented as cystic edema. Therefore, the visual acuity of patients with cystoid macular edema is often weaker than that of healthy people of the same age, as cystic edema can affect glial components and destroy photoreceptor cells, which when severe, may cause blindness (14). According to the different pathological types of leakage, macular edema occurs in both the inner nuclear layer and the outer plexiform layer; therefore, the edema layer can be divided into three types: inner nuclear layer, outer plexiform layer, and mixed type. However, the three types of edema also have their own characteristics. For example, the BCVA of mixed and outer retinal edema is lower than that of inner layer edema. Previous studies have shown that blood components and wastes in the vesicles-like space may affect cell metabolism and cause changes in osmotic pressure, resulting in stretched axons and disfunction of bipolar cells with decreased photosensitivity and signal transduction (19). Similarly, photoreceptor cells are stressed and a similar cascade of changes occurs, leading to a decline in photographic function. In other studies, the vesicles with high density are mostly located in outer retinal edema since the component of the high density point is lipoprotein. Lipoproteins penetrate into the ELM layer, and the lipoproteins passing through the ELM layer can also destroy the IS/OS layer to directly induce photoreceptor degeneration (5). In DRME, there are inflammatory factors that lead to the destruction of the outer barrier, and then the edema of the outer retina is aggravated, and photoreceptor cells are damaged. In addition, the outer retinal edema is anatomically close to the IS/OS layer and ELM layer; therefore, edema can increase intracapsular pressure physically and further aggravate inner retinal edema. These factors reduce the BCVA visual acuity of outer retinal edema. Unlike outer and inner retinal edema, due to the presence of both inner and outer retinal edema, mixed retinal edema further enhances the edema effect and further reduces BCVA.

There are some limitations in this study, however; for example, the sample size was small, BCVA was not measured based on ETDRS, and it was difficult to exclude the influence of other factors on BCVA or retinal dysfunction. Therefore, it is still necessary to assess whether the retinal function is abnormal with the help of an electroretinogram. This study performed OCT scans of the macular area in four directions of 0, 45, 90, and 135 degrees in 64 eyes, and did not perform more refined scans, such as 8- or 16-equivalent scans. And the study area was only 1 mm as the radius and fovea as the center, and no larger area study was done. In addition, imaging with OCT scans in DME may have an impact on the imaging of the outer microstructure when there is hard exudate or hemorrhagic occlusion in the retina, leading to measurement errors.

In summary, according to OCT examination, the CFT is small, the ELM layer and IS/OS layer are relatively integral, and BCVA is closely correlated with inner retinal edema in DRME.

## Data Availability Statement

The original contributions presented in the study are included in the article/supplementary material. Further inquiries can be directed to the corresponding author.

## Ethics Statement

The studies involving human participants were reviewed and approved by the ethics committee of The First Affiliated Hospital of China Medical University. The patients/participants provided their written informed consent to participate in this study.

## Author Contributions

SL and LC conceived the idea and conceptualized the study. RH collected the data. ZJ and LH analyzed the data. SL and LC drafted the manuscript. SL and LC reviewed the manuscript. All authors read and approved the final draft.

## Funding

Project name: Basic Research Project of Heilongjiang Provincial Education Department. No. 2019-KYYWF1354.

## Conflict of Interest

The authors declare that the research was conducted in the absence of any commercial or financial relationships that could be construed as a potential conflict of interest.

## Publisher’s Note

All claims expressed in this article are solely those of the authors and do not necessarily represent those of their affiliated organizations, or those of the publisher, the editors and the reviewers. Any product that may be evaluated in this article, or claim that may be made by its manufacturer, is not guaranteed or endorsed by the publisher.
